# Developing a geographical–meteorological indicator system and evaluating prediction models for alveolar echinococcosis in China

**DOI:** 10.1038/s41370-024-00664-z

**Published:** 2024-04-23

**Authors:** Chuizhao Xue, Baixue Liu, Yan Kui, Weiping Wu, Xiaonong Zhou, Ning Xiao, Shuai Han, Canjun Zheng

**Affiliations:** 1 National Institute of Parasitic Diseases, Chinese Center for Disease Control and Prevention (Chinese Center for Tropical Diseases Research), National Key Laboratory of Intelligent Tracking and Forecasting for Infectious Diseases, Key Laboratory on Parasite and Vector Biology of Ministry of Health, WHO Centre for Tropical Diseases, National Center for International Research on Tropical Diseases of Ministry of Science and Technology, Shanghai, China, 207, Ruijin Er Road, Huangpu District, Shanghai, 200025 China; 2 Chinese Center for Disease Control and Prevention, Beijing, China, 155, Changbai Road, Changping District, Beijing, 102206 China

**Keywords:** Alveolar echinococcosis, Geo-meteorological factors, Indicator system, Modeling.

## Abstract

**Background:**

Geographical and meteorological factors have been reported to influence the prevalence of echinococcosis, but there’s a lack of indicator system and model.

**Objective:**

To provide further insight into the impact of geographical and meteorological factors on AE prevalence and establish a theoretical basis for prevention and control.

**Methods:**

Principal component and regression analysis were used to screen and establish a three-level indicator system. Relative weights were examined to determine the impact of each indicator, and five mathematical models were compared to identify the best predictive model for AE epidemic levels.

**Results:**

By analyzing the data downloaded from the China Meteorological Data Service Center and Geospatial Data Cloud, we established the KCBIS, including 50 basic indicators which could be directly obtained online, 15 characteristic indicators which were linear combination of the basic indicators and showed a linear relationship with AE epidemic, and 8 key indicators which were characteristic indicators with a clearer relationships and fewer mixed effects. The relative weight analysis revealed that monthly precipitation, monthly cold days, the difference between negative and positive temperature anomalies, basic air temperature conditions, altitude, the difference between positive and negative atmospheric pressure anomalies, monthy extremely hot days, and monthly fresh breeze days were correlated with the natural logarithm of AE prevalence, with sequential decreases in their relative weights. The multinomial logistic regression model was the best predictor at epidemic levels 1, 3, 5, and 6, whereas the CART model was the best predictor at epidemic levels 2, 4, and 5.

## Introduction

Echinococcosis, also known as hydatid disease, is caused by *Echinococcus* spp. and has been regarded as one of the most important parasitic zoonotic diseases worldwide [1–17]. Among the major species identified *Echinococcus granulosus* and *Echinococcus multilocularis* pose a substantial threat to public health, respectively leading to cystic echinococcosis (CE) and alveolar echinococcosis (AE). China bears the highest global burden of echinococcosis worldwide [18], with a higher prevalence of both CE and AE in the north and northwest regions compared to other areas [17]. According to the former research, there were approximately 18,235 (CIS 11,900–28,200) new cases of AE per annum globally with 16,629 (91%) occurring in China [19].

Previous studies have demonstrated that various geographical and meteorological factors can impact the transmission of echinococcosis by affecting host distribution and exposed egg activity. It is relatively easy for humans to control the transmission of Echinococcus in residential areas, but is very difficult to affect the transmission between host animals in the wild. Therefore, the impact of geographical and meteorological factors on the distribution and survival of animal hosts and eggs is greater in the wilderness, in turn leading to a greater impact on transmission. For example, field studies have reported poor survival of *E. multilocularis* eggs at temperatures >25 °C and following exposure to extreme cold conditions (≤−83 °C), whereas temperatures of –18 °C to 4 °C are well-tolerated [20]. In the same study, the authors reported that *E. multilocularis* eggs are also sensitive to different levels of moisture and humidity [20]. Other studies have reported a considerable correlation between mean annual air temperature and the prevalence of *E. multilocularis* in red foxes, as well as a correlation between prevalence and mean annual precipitation [21].

Further, when examined as independent variables, grassland area ratio and land surface temperature have been shown to exhibit positive and negative correlations with the prevalence of human CE, respectively [22]. By combining maps of environmental and biological covariates with information concerning known cases of human CE in the Xizang Autonomous Region of China, researchers have reported significant associations between CE prevalence and annual average precipitation, elevation, and water accessibility (*P* < 0.05) [23]. However, another previous study conducted in Xizang reported that minimum temperature, maximum temperature, relative humidity, precipitation, terrain, land use, and normalized difference vegetation index (NDVI) had little impact on the prevalence of AE, although the interactions between them enhanced their separate effects on AE prevalence [24]. Despite this finding, few studies overall have examined geographical and meteorological risk factors for AE in China.

To provide further insight into the impact of geographical and meteorological factors on AE prevalence and establish a theoretical basis for appropriate prevention and control measures, we conducted an exploratory analysis of these factors across all six affected provinces in China, based on an epidemiological survey conducted from 2012 to 2016: the Xizang Autonomous Region, Qinghai Province, Sichuan Province, Gansu Province, Xinjiang Uygur Autonomous Region, and Ningxia Hui Autonomous Region [17].

## Materials and methods

### Collection and processing of population infection data

The county-level prevalence data of AE from 2012 to 2016 were collected from recent scientific papers and reports [17, 25]. The same methodology was used for all the epidemiological surveys carried out in these counties, and the details of the diagnosed cases and human prevalence estimates are described in Wu and Zheng [17, 25].

### Sources of geographical/meteorological data and processing

Echinococcosis is a chronic disease that is difficult to detect during its initial stage, therefore the mean value from 1981 to 2010 of the meteorological variables of county-level administrative regions were used for analyzing. Surface meteorological data from 1981 to 2010 (ID:1.2.156.416. CMA. D3. A002.001. OB. WB. CHN. MUL. MON. ZD. 1) were downloaded from the China Meteorological Data Service Center (http://data.cma.cn/), including 50 variables related to pressure, temperature, relative humidity, precipitation, and wind at all meteorological stations in the provinces with echinococcosis prevalence (Supplementary Table 1).

SRTMDEM and MODIS 500M monthly synthetic NDVI data for 2000 to 2015 were downloaded from the Geospatial Data Cloud (https://www.gscloud.cn/).

### Statistical analysis and modeling

R-4.1.2 (R Foundation for Statistical Computing, Vienna, Austria) was used for statistical analysis. The base and dplyr packages were used for data cleaning, while the krige function of the gstat package was used for Kriging spatial interpolation. The base and dplyr packages were used to calculate the mean values of NDVI, altitude (DEM), and meteorological data. The principal function of the psych package was used to perform principal component analysis, and the lm function of the stats package was used to perform one-way linear regression analysis. The cv.glmnet and glmnet functions of the glmnet package were used to establish a least absolute shrinkage and selection operator (LASSO) regression model, while the lm function of the stats package was used to establish a multivariable linear regression model. The step function of the stats package was used to establish a stepwise regression model. The multinom function of the nnet package was used to establish a multinomial logistic regression model. The lda function of the MASS package was used to establish a Bayesian discriminant model, and the rpart function of the rpart package was used to establish a Classification and Regression Tree Model (CART). Relative weight analysis was also performed using the rwa package. Map created using the Free and Open Source QGIS-3.22.14 (Open Source Geospatial Foundation). The significance level is 0.05.

### Epidemic levels for Echinococcus multilocularis

Using the k-means function in the statistics rwa package, AE epidemic level was classified based on the natural logarithm of the prevalence rate. Classification performance was evaluated using the goodness of classification. The minimum number of clusters with a goodness of classification >95% was selected as the optimal number of prevalence levels, as follows:

$${Goodness}\,{of}\,{classification}=\mathop{\sum }\limits_{k=1}^{k}\mathop{\sum }\limits_{j=1}^{{n}_{k}}{\left({x}_{k,j}-\bar{{x}_{k,\cdot }}\right)}^{2}/\mathop{\sum }\limits_{i}^{n}{\left({x}_{i}-\bar{x}\right)}^{2}$$

where *k* is the number of cluster centers, ${n}_{k}$ is the number of observations in cluster *k*, and *n* is the total number of observations.

### Indicator library

NDVI, DEM, and all the meteorological variables were used as basic indicator library. To establish a composite indicator library, the principal components of atmospheric pressure, temperature, precipitation, and wind were extracted from 49 climatic variables, and the significance of each principal component was analyzed. The single variable indicators were NDVI, DEM, and relative humidity, which were labeled as composite indicators. Effective composite indicators that had a linear relationship with the natural logarithm of the AE prevalence rate were selected via single-factor linear regression of all composite indicators.

Key indicators were selected based on the λ with the smallest mean error via 10-fold cross-validation, and a multivariate LASSO regression model was established using the λ. Composite indicators with non-zero coefficients were selected as key indicators.

### Establishment of a three-level indicator system

The Key-Characteristic-Basic Indicators System(KCBIS) was Established based on the results of principal components analysis and mathematical models. The key indicators were defined as effective composite indicators with a non-zero coefficients in the LASSO regression model. Define effective composite indicators selected in 2.5 part using single-factor linear regression models as characteristic indicators. Basic indicators were defined as those who linearly combined to create an effective composite indicator. On the other hand, characteristic indicators are linear combinations of basic indicators, and the combination method is based on the results of principal component analysis which described in 2.5 part.

### Analysis of relative weights for key indicators

The relative weights of the key indicators were analyzed using the rwa package and RWA function in R-4.1.2.

### Establishment and comparison of multiple models

Using the natural logarithm of AE prevalence rate as the dependent variable and the key indicators as independent variables, we established a multivariable linear regression model and stepwise regression model. Using the prevalence level as the dependent variable and the key indicators as independent variables, we also established a naive Bayesian classification model using the proportion of each prevalence category as the a priori weight. Using the prevalence level as the dependent variable and the key indicators as independent variables, we further established multinomial logistic regression and CART models.

All samples were used to predict the prevalence rate or level. Model performance was evaluated by calculating and comparing accuracy, precision, sensitivity, specificity, and the F1-Score.

## Results

### Composite indicators of atmospheric pressure

**Fig. 1:**
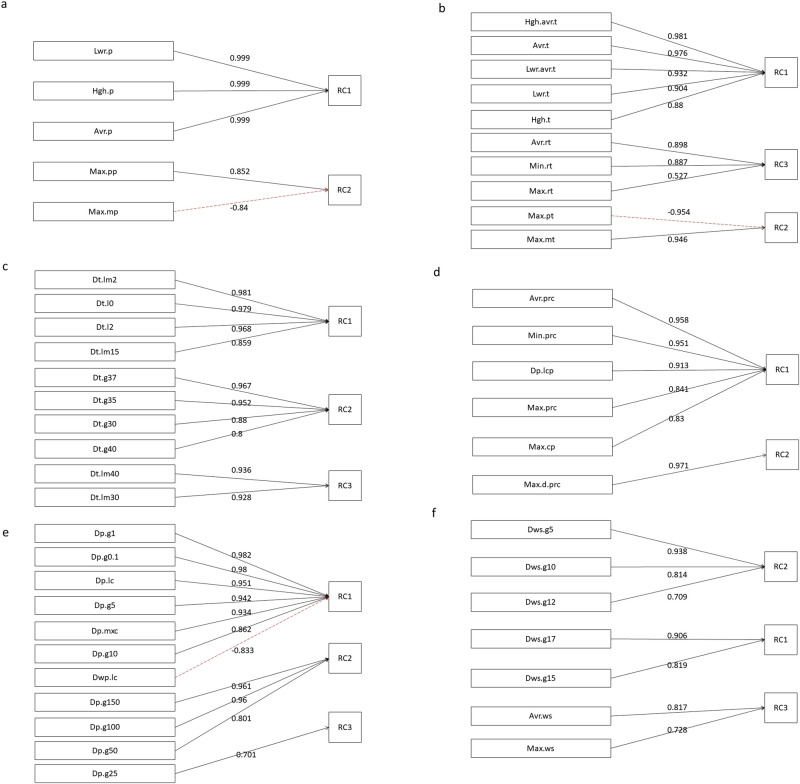
Component analysis of meteorological factors. **a**–**f** represents the principal component analysis of atmospheric pressure factors, temperature measurement factors, temperature duration factors, precipitation measurement factors, precipitation duration factors, wind factors.

The principal component analysis of the basic indicators of atmospheric pressure revealed that two composite indicators could explain more than 90% of the variance in the original five basic indicators (Supplementary Table 2 and Fig. 1a). RC1 represented the basic atmospheric pressure condition, recorded as atoms_Val, while RC2 represented the difference between the positive and negative atmospheric pressure anomalies, recorded as atmos_an.

### Composite indicators of temperature

The basic temperature indicators were divided into two categories. The first category included indicators of temperature measurements, with three composite indicators extracted via the principal component analysis(Supplementary Table 2 and Fig. 1b). In this analysis, RC1 represented the basic temperature condition, recorded as temp_Val, RC2 represented the difference between the negative and positive temperature anomalies, recorded as temp_anom, and RC3 represented the daily temperature range, recorded as temp_DRg. The second category included temperature duration indicators, with three composite indicators extracted via the principal component analysis(Supplementary Table 2 and Fig. 1c). In this analysis, RC1 represented the duration index of cold days monthly, recorded as CldD (i.e., the comprehensive index of days with temperatures not exceeding 2 °C, 0 °C, –2 °C, and –15 °C). Similarly, RC2 represented the duration index of extremely hot days monthly, recorded as ExtrHtD (i.e., the comprehensive index of days with temperatures not less than 30 °C, 35 °C, 37 °C, and 40 °C). Lastly, RC3 represented the duration index of extremely cold days monthly, recorded as ExtrCldD and reflecting the comprehensive index of days with temperatures not exceeding –30 °C and –40 °C.

### Composite indicators of precipitation

The basic indicators of precipitation were divided into two categories. The first category included precipitation measurements, with two composite indicators extracted using principal component analysis (Supplementary Table 2 and Fig. 1d). In this analysis, RC1 represented the monthly precipitation index, recorded as prec_Val_M, while RC2 represented the daily precipitation index, recorded as prec_Val_D. The second category included indicators of precipitation duration, with three composite indicators extracted using principal component analysis(Supplementary Table 2 and Fig. 1e). In this analysis, RC1 represented the duration index of monthly rainy days, recorded as Rainy_Ds and reflecting the comprehensive index of rainy days. RC2 represented the monthly rainstorm days index, recorded as rainform_Ds and reflecting the comprehensive index of days with daily precipitation levels not less than 50 mm, 100 mm, and 150 mm. RC3 represented heavy rain days index monthly, recorded as Hv_Rny_Ds and reflecting the comprehensive index of days with daily precipitation of not less than 25 mm.

### Composite wind indicators

In the principal component analysis, three composite indicators could be extracted from the basic indicators of wind factors (Supplementary Table 2 and Fig. 1f). In this analysis, RC1 represented the fresh breeze days index monthly, recorded as Fsh_brz_Ds and reflecting the comprehensive index of days with wind speeds of not less than 15 m/s and 17 m/s. RC2 represented the monthly gentle breeze days index, recorded as Gt_brz_Ds and reflecting the comprehensive index of days with wind speeds not less than 5 m/s, 10 m/s, and 12 m/s. RC3 represented the basis of wind speed, recorded as Wnd_spd_val and reflecting the comprehensive index of average and maximum wind speeds.

### Effective composite indicators and characteristic indicators

All the composite indicators mentioned above are of particular realistic significance (Table 1). The single-factor linear regression analysis revealed a significant linear relationship between the natural logarithm of AE prevalence and the following, which were thus identified as effective composite indicators: atoms_Val, atmos_an, temp_Val, temp_anom, CldD, ExtrHtD, ExtrCldD, prec_Val_M, prec_Val_D, Rainy_Ds, rainstorm_Ds, Gt_brz_Ds, Fsh_brz_Ds, Wnd_spd_val, DEM respectively (Table 2). Among these, atoms_Val, atmos_an, temp_Val, and ExtrHtD were negatively correlated with the natural logarithm of AE prevalence. In contrast, temp_anom, ExtrCldD, prec_Val_M, prec_Val_D, Rainy_Ds, rainstorm_Ds, Gt_brz_Ds, Fsh_brz_Ds, Wnd_spd_Val, and DEM were positively correlated with the natural logarithm of AE prevalence (Table 2, Supplementary Fig. 1, Supplementary Fig. 2). These 15 effective composite indicators were used as characteristic indicators.

**Table 1:** The meanings of the composite indicators.

Indicators	Meanings
atoms_Val	The basic atmospheric pressure condition
atmos_an	The difference between the positive and negative atmospheric pressure anomalies
temp_Val	The basic temperature condition
temp_anom	The difference between the negative and positive temperature anomalies
temp_DRg	The daily temperature range
CldD	The duration index of cold days monthly
ExtrHtD	The duration index of extremely hot days monthly
ExtrCldD	The duration index of extremely cold days monthly
prec_Val_M	The monthly precipitation index
prec_Val_D	The daily precipitation index
Rainy_Ds	The duration index of monthly rainy days
rainstorm_Ds	The monthly rainstorm days index
Hv_Rny_Ds	The heavy rain days index monthly
Fsh_brz_Ds	The fresh breeze(speed ≥15 m/s) days index monthly
Gt_brz_Ds	The monthly gentle breeze(speed ≥5 m/s) days index
Wnd_spd_val	The basis of wind speed
RH	Monthly average relative humidity multi-years
NDVI	The mean of normalized difference vegetation index
DEM	The mean of the elevation of Digital Elevation Model

**Table 2:** Single-factor linear regression analysis.

Dependent variable	Independent variable	Coefficients					Multiple R-squared	Adjusted R-squared	*F*-test	
		Estimate	Std. Error	*t* value	*p*-value	Signif. codes			*F*	*p*-value
ln(pr)	atoms_Val	−0.0033	0.0004	−7.9150	0.0000	***	0.392	0.386	62.640	0.0000
	atmos_an	−0.0946	0.0118	−8.0170	0.0000	***	0.399	0.392	64.270	0.0000
	temp_Val	−0.0513	0.0076	−6.7270	0.0000	***	0.318	0.311	45.250	0.0000
	temp_anom	0.1732	0.0222	7.7990	0.0000	***	0.385	0.379	60.820	0.0000
	temp_DRg	−0.0666	0.0552	−1.2060	0.2310		0.015	0.005	1.455	0.2306
	CldD	0.0605	0.0094	6.4170	0.0000	***	0.298	0.291	41.170	0.0000
	ExtrHtD	−0.2845	0.0406	−7.0100	0.0000	***	0.336	0.329	49.140	0.0000
	ExtrCldD	0.2497	0.0627	3.9860	0.0001	***	0.141	0.132	15.890	0.0001
	prec_Val_M	0.0060	0.0020	3.2530	0.0020	**	0.098	0.089	10.590	0.0016
	prec_Val_D	0.0100	0.0040	2.5290	0.0130	*	0.062	0.052	6.395	0.0131
	Rainy_Ds	0.0130	0.0040	3.0050	0.0030	**	0.085	0.076	9.028	0.0034
	rainstorm_Ds	0.0690	0.0290	2.3440	0.0210	*	0.054	0.044	5.495	0.0211
	Hv_Rny_Ds	−0.0310	0.0310	−0.9940	0.3230		0.010	0.000	0.988	0.3227
	Gt_brz_Ds	0.1070	0.0180	6.0150	0.0000	***	0.272	0.264	36.170	0.0000
	Fsh_brz_Ds	0.2200	0.0430	5.0920	0.0000	***	0.211	0.203	25.930	0.0000
	Wnd_spd_val	0.2250	0.0440	5.1510	0.0000	***	0.215	0.207	26.530	0.0000
	RH	−0.0120	0.0210	−0.5800	0.5640		0.003	−0.007	0.336	0.5636
	DEM	0.0010	0.0000	7.0060	0.0000	***	0.336	0.329	49.080	0.0000
	NDVI	0.4400	1.2020	0.3660	0.7150		0.001	−0.009	0.134	0.7150

*pr* represents prevalence.

***p < 0.001, **p < 0.01, *p < 0.05.

### Key indicators

In the 10-fold cross-validation, the λ corresponding to the minimum of the mean error of the LASSO regression was 0.01484477 (Supplementary Fig. 3). In the LASSO regression model based on λ, the variables with non-zero coefficients were atmos_an, temp_Val, temp_anom, CldD, ExtrHtD, prec_Val_M, Fsh_brz_Ds, and DEM, all of which were used as key indicators (Table 3).

**Table 3:** Coefficients of the LASSO Regression.

Dependent variable	Independent variable	Coefficients
ln(pr)	(Intercept)	−6.2113
	atoms_Val	.
	atmos_an	−0.0098
	temp_Val	−0.0055
	temp_anom	0.0620
	CldD	0.0492
	ExtrHtD	0.1139
	ExtrCldD	.
	prec_Val_M	0.0083
	prec_Val_D	.
	Rainy_Ds	.
	rainstorm_Ds	.
	Gt_brz_Ds	.
	Fsh_brz_Ds	−0.0246
	Wnd_spd_val	.
	DEM	0.0002

· Coefficients are 0.

### Establishment of a three-level indicator system

By calculating and screening the key indicators, characteristic indicators, and related basic indicators, we established a three-level indicator system. This Key-Characteristic-Basic Indicator System (KCBIS) included 50 basic indicators, 15 characteristic indicators, and 8 key indicators (Supplementary Fig. 4). Obviously, the 50 basic indicators could be directly observed at meteorological stations, the 15 characteristic indicators could be produced by linear combination of the basic indicators and showed a linear relationship with AE epidemic, and the 8 key indicators were characteristic indicators with clearer relationships and fewer mixed effects.

### Analysis of relative weights for key indicators

**Fig. 2:**
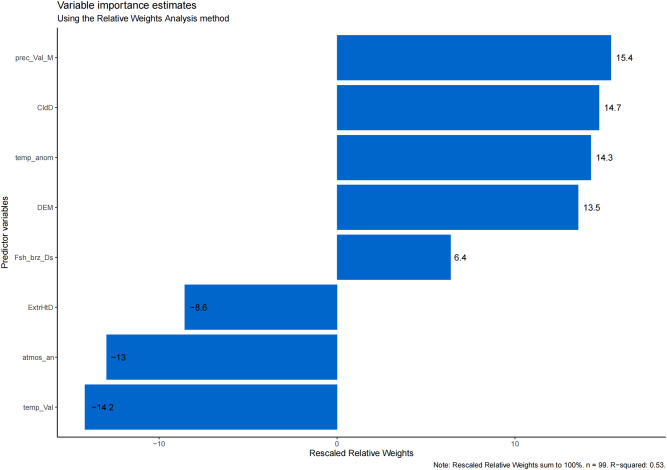
The result of analysis of relative weights for key indicators.

In the analysis of relative weights for key indicators, we observed a positive correlation between the natural logarithm of AE prevalence and the following, with sequential decreases in the relative weight of each: prec_Val_M, CldD, temp_anom, DEM, Fsh_brz_Ds. Similarly, we observed negative correlations between the natural logarithm of AE prevalence and temp_Val, atoms_an, and ExtrHtD, again with sequential decreases in the relative weight of each (Fig. 2).

### Classification of epidemic levels for *Echinococcus multilocularis*

When divided into 6 categories based on the natural logarithm of AE prevalence, prevalence (levels 1–6), the goodness of classification for R^2^ reached over 95% (Supplementary Fig. 5). The AE prevalence rates represented by levels 1~6 are ~0.01%, ~0.05%, ~0.15%, ~0.38%, ~1.73%, and 1.73%~, respectively (Supplementary Table 3).

### Model establishment and comparison

The multivariable linear regression model and the stepwise regression model, the multinomial logistic regression model, the naive Bayesian classification model, and the CART model was established (the parameters showed in Supplementary Tables 4, 5, 6, and Supplementary Fig. 6 respectively). Among the models compared, the CART model had the highest accuracy, sensitivity, and specificity values, and the multinomial logistic regression model had the highest precision value and F1-Score. According to accuracy and F1-Score, the best predictor for levels 1, 3, 5, and 6 was the multinomial logistic regression model, while the best predictor for levels 2, 4, and 5 was the CART model (Table 4).

**Table 4:** Comparison of model performance.

Model	Level	Accuracy	Precision	Sensitivity	Specificity	F1-Score
Multivariable linear regression model	1	0.00	-	0.00	0.97	-
	2	0.24	0.42	0.36	0.67	0.39
	3	0.09	0.14	0.19	0.65	0.16
	4	0.26	0.40	0.41	0.53	0.41
	5	0.37	0.50	0.58	0.62	0.54
	6	0.00	-	0.00	0.95	-
	Total^a^	0.37	0.35	0.37	0.63	0.36
Stepwise regression model	1	0.00	-	0.00	0.97	-
	2	0.24	0.40	0.36	0.66	0.38
	3	0.03	0.05	0.06	0.66	0.06
	4	0.27	0.41	0.45	0.52	0.43
	5	0.33	0.46	0.54	0.61	0.50
	6	0.00	-	0.00	0.95	-
	Total^a^	0.35	0.33	0.35	0.63	0.34
Multinomial logistics regression model	1	1.00	1.00	1.00	0.97	1.00
	2	0.48	0.63	0.68	0.69	0.65
	3	0.13	0.30	0.19	0.77	0.23
	4	0.38	0.58	0.52	0.60	0.55
	5	0.50	0.60	0.75	0.64	0.67
	6	0.57	0.67	0.80	0.93	0.73
	Total^a^	0.59	0.57	0.59	0.68	0.58
Naive Bayesian classification model	1	0.33	0.40	0.67	0.94	0.50
	2	0.52	0.68	0.68	0.71	0.68
	3	0.08	0.18	0.13	0.75	0.15
	4	0.31	0.47	0.48	0.55	0.47
	5	0.37	0.54	0.54	0.65	0.54
	6	0.50	0.57	0.80	0.92	0.67
	Total^a^	0.51	0.49	0.51	0.67	0.50
Classification and regression tree model	1	0.38	0.38	1.00	0.92	0.55
	2	0.64	0.75	0.82	0.72	0.78
	3	0.00	-	0.00	0.84	-
	4	0.50	0.62	0.72	0.58	0.67
	5	0.50	0.58	0.79	0.62	0.67
	6	0.00	-	0.00	0.95	-
	Total^a^	0.62	0.50	0.62	0.69	0.55

^a^Precision, Sensitivity, and Specificity represent the weighted precision, weighted sensitivity, and weighted specificity based on the proportion of classes.

## Discussion

In this study, we established a geographical–meteorological indicator system named Key-Characteristic-Basic Indicators System(KCBIS) to identify which factors exert a significant impact on the prevalence of AE in China, based on the results of regression and relative weight analyses. The KCBIS included 50 basic indicators, 15 characteristic indicators, and 8 key indicators.

The basic indicators included altitude (DEM) and 49 meteorological indicators, all of which can be directly measured at meteorological stations in China, making them easy to obtain. Different basic indicators can reflect a certain meteorological or geographical phenomenon to a certain extent, respectively. It should be emphasized here that echinococcosis is a chronic disease that is not easily detected in the early stages. Only when the lesion reaches a certain size can it be determined through imaging methods(B-ultrasound or computed X-ray tomography). Therefore, the basic indicators used in this study are not simple observation values of that year, but the average value of 30 years before the year of AE epidemic data. This study used geographic and meteorological data from 1981 to 2010 to analyze the AE epidemic from 2012 to 2016. Inevitably, there will be a very small number of cases infected before 1981 or after 2010, which will have a subtle impact on the results of this study. To solve this problem, it is necessary to conduct long-term monitoring of population infections and geographical or meteorological changes in epidemic areas, which is our further research direction.

The relationship between different geographical and meteorological data can seriously affect data analysis, such as the collinearity between factors (temperature decreases linearly with altitude to a certain extent) and within factors (certain linear relationship between average daily precipitation and maximum or minimum daily precipitation). If these single indicators are directly screened and then one or some indicators of a certain factor are roughly deleted, a large amount of information in that factor is often lost. Based on observations of different factors, most of them have several indicators, which could be classified to 2 or 3 dimensions such as basic conditions, variation, and duration. Then, principal component analysis is used to extract principal components from different dimensions, replacing the original indicators. On the one hand, this can reduce collinearity while retaining as much information as possible about the factor, and on the other hand, it can more clearly reflect the characteristics of the indicator from different dimensions. This is the basic idea of how we use basic indicators to generate a comprehensive indicator, from which we screened the characteristic indicators. So, from a mathematical perspective, the characteristic indicators were linear combinations of the basic indicators. The characteristic indicator could be regarded as a representation of a certain meteorological or geographical phenomenon, eliminating the collinearity that exists when observing the meteorological phenomenon at different scales. There is a significant correlation between the characteristic indicators and the epidemic of AE, showed the characteristic indicators had reference sense in indicating the strength of local AE prevalence. When evaluating the impact of individual geographical and meteorological phenomenon on the epidemic of AE, full consideration should be given to each characteristic indicators.

The key indicators were those characteristic indicators with significant influence, which were used in the main analyses and to develop the predictive models. The key indicators further reduced the collinearity between observations of different geographical and meteorological phenomena on the basis of characteristic indicators, and eliminated the mixed effect of different geographical and meteorological phenomena on the epidemic of AE to some extent, so that the key indicators has stronger indication and pertinence during the modeling process.

The relative weight analysis also revealed sequential decreases for the correlation between the natural logarithm of AE prevalence and prec_Val_M, CldD, temp_anom, temp_Val, DEM, atmos_an, ExtrHtD, and Fsh_brz_Ds.

Previous studies have found that different levels of water or humidity can affect [20] or interact with other environmental factors [23, 24] to affect the activity of *Echinococcus* eggs or disease transmission, reporting significant correlations between disease incidence and annual average precipitation [5]. Our study further identified a significant positive correlation between characteristic indicators of monthly precipitation (prec_Val_M) and the natural logarithm of AE prevalence.

Research has demonstrated that Echinococcus eggs can tolerate temperatures ranging from –18 °C to 4 °C [20], and a significant correlation between annual average temperature and rates of *Echinococcus multilocularis* infection has been observed in red foxes [5]. Another study have revealed that minimum temperature and maximum temperature have little effect on the incidence of AE [24]. However, in the current study, the characteristic indicator representing the basic temperature condition (temp_Val) and the characteristic indicator representing the number of extremely hot days each month (ExtrHtD) were significantly negatively correlated with the natural logarithm of AE prevalence. While the characteristic indicators representing the index of cold days (CldD) presented significant positive correlations with the natural logarithm of the AE prevalence rate, consistent with previous study on the temperature tolerance of eggs [20]. The difference between the negative and positive temperature anomalies (temp_anom) exhibited significant positive correlations with the natural logarithm of the AE prevalence rate, further supporting the notion that the minimum and highest temperatures can enhance the impact of other factors on AE prevalence rates [24]. Further, relative weights were higher for the characteristic indicators with positive effects than for those with negative effects.

Although high rates of AE prevalence in China have mainly been reported in the Qinghai-Tibet Plateau, the relationship between DEM and the incidence of AE is not a simple linear one. In the current study, the DEM exhibited a significant positive correlation with the natural logarithm of AE prevalence. It is well known that increases in altitude are associated with decreases in atmospheric pressure. However, in our study, the change of atmospheric pressure was a more significant indicator of AE prevalence compared with the atmospheric pressure itself. Specifically, the difference between positive and negative atmospheric pressure anomalies(atmos_an) was negatively correlated with the natural logarithm AE prevalence.

Our analysis identified a weak positive correlation between the characteristic indicator of days of fresh breeze(wind speed ≥15 m/s) and AE prevalence levels. There are currently very few studies reporting the relationship between wind and prevalence. However, the wind can affect egg dispersal and vapor evaporation, which in turn affects the environment risk and ultimately leads to changes in prevalence. Further studies are required to determine whether this indicator influences AE prevalence via humidity, egg dispersal, or other factors.

In this research, 5 models were developed using the key indicators and AE epidemic levels, and be compared using accuracy and the F1-Score. Compared with other research [26], what goes further is we assume that the impact of each variable varies at different epidemic levels and attempt to find well-performing models for each level. The results indicate that no model outperforms other models at all levels (Level 1~Level 6). Overall, the accuracy and F1-Score were highest for the multinomial logistic regression model and CART model. The multinomial logistic regression model was the best predictor of epidemic levels 1, 3, 5, and 6, whereas the CART model was the best predictor of epidemic levels 2, 4, and 5. This indicates that the influence of various factors is not constant at different epidemic levels and that a combination of multiple segmented models must be considered in future studies. It is also possible that some factors only begin to exert a certain effect when other factors are considered or when their weights reach a certain threshold which needs to be further researched. Although the accuracies and F1-Scores of the multinomial logistic regression model and CART model were same at epidemic level 5, the precision of the multinomial logistic regression model is higher while the sensitivity of CART model is higher. Complex indicators often lead to overfitting of the model. To avoid this phenomenon, we have taken a series of measures. Firstly, when selecting key indicators (independent variables), LASSO regression was used, which introduced regularization penalty terms, and a 10-fold cross-validation method was used to estimate regularization parameters. Secondly, using the large sample size, the prevalence data in this study was obtained from the largest epidemiological survey of echinococcosis in the world to date. Thirdly, choose models that have already been applied in published studies or other simple models that have not yet been applied in echinococcosis prediction. Echinococcosis is a chronic disease with insignificant early symptoms. Although the exact infection time of cases is unknown, it is generally within 30 years before the B-ultrasound examination. So the proportion of cases infected before 1981 or after 2010 must be very low. In addition, the AE prevalence data used in this study was not obtained from the overall population, but was credible due to the scientific sampling method. Therefore, although there may be some bias, it does not affect the validity of the results of this study.

At present, China’s control measures for livestock and dogs are very effective, and the next focus is on the prevention and control of wild animal hosts. However, the field investigation of the transmission cycle in the wild is very difficult. This study can, to a certain extent, conduct risk assessment on vast outdoor areas with unknown epidemic levels through geographic and meteorological models. Combined with sampling verification, high-risk areas for *Echinococcus multilocularis* in the wild can be identified, and precise prevention and control measures such as deworming wild canines can be carried out. High-risk areas could be marked to issue warnings to nomadic populations. Focus on people and dogs returning from nomadic grazing with high-risk, carry out targeted screening and dogs deworming. The future research should consider the combination of multiple segmented models, while focusing on evaluating the impact of geographic and meteorological factors in some special habitats, in order to further improve and optimize the model. The effectiveness of geo-meteorological models is reflected in the fact that geo-meteorological factors can significantly affect the infection of human echinococcosis by affecting the ecological distribution of animal hosts and the activity of pathogens. It has significant reference value for other countries or regions which are similar to the epidemic areas of AE in China, such as vast animal husbandry areas with grasslands, mountains, and cold weather, and is also applicable to the areas where human infections are mainly caused by transmission cycles composed of dogs, foxes, wolves, and rodents. This study also provides a feasible reference method for the establishment of other geo-meteorological prediction models for infectious diseases transmitted through animal hosts or vectors.

The Intelligent Prediction Large Model (IPLM) triggered by multiple points is one of the important development directions for future disease monitoring and early warning. As one of the important foundations of IPLM, the combination of geographic meteorological models with animal index system models, human behavior system models, and socio-economic system models can effectively improve the effectiveness of IPLM.

## Conclusion

In this study, we established a Key-Characteristic-Basic Indicators System(KCBIS) of 50 basic, 15 characteristic, and 8 key geographical and meteorological indicators exerting a significant impact on AE epidemic levels. The 50 basic indicators could be directly observed at meteorological stations, the 15 characteristic indicators could be produced by linear combination of the basic indicators and showed a linear relationship with AE epidemic, and the 8 key indicators were characteristic indicators with a clearer relationships and fewer mixed effects. Comparison of five mathematical models revealed that the influence of various factors was not constant across different epidemic levels. The best predictor at epidemic levels 1, 3, 5, and 6 was the multinomial logistic regression model, whereas the best predictor at epidemic levels 2, 4, and 5 was the CART model. Thus, future studies should consider a combination of multiple segmented models.

## Supplementary information

Supplementary Tables and Legends of Supplementary Figures

Supplementary Figure 1

Supplementary Figure 2

Supplementary Figure 3

Supplementary Figure 4

Supplementary Figure 5

Supplementary Figure 6

## Data Availability

The data that support the findings of this study are available from the author of the study (Mr. Chuizhao Xue) but restrictions apply to the availability of these data, which are not publicly available. Data are however available from the authors upon reasonable request and with permission of the National Institute of Parasitic Diseases, Chinese Center for Disease Control and Prevention (Chinese Center for Tropical Diseases Research).
