# Supplementary material for: Developing a geographical–meteorological indicator system and evaluating prediction models for alveolar echinococcosis in China
Source: J Expo Sci Environ Epidemiol. 2024 Apr 23;35(2):254–63. doi: 10.1038/s41370-024-00664-z (PMC12009731; doi:10.1038/s41370-024-00664-z)
Supplement: Supplementary file 1 — Supplementary Tables and Legends of Supplementary Figures [file 41370_2024_664_MOESM1_ESM.docx]

**Supplementary Table 1. Meteorological factors, variables, and meanings**

| Meteorological factors | Variable | Meaning |
| --- | --- | --- |
| Pressure | Avr.p | Monthly average pressure of the station multi-years |
|  | Hgh.p | Monthly highest pressure of the station multi-years |
|  | Lwr.p | Monthly lowest pressure of the station multi-years |
|  | Max.pp | Monthly maximum positive departure of pressure multi-years |
|  | Max.mp | Monthly maximum minus departure of pressure multi-years |
| Temperature | Avr.t | Monthly average temperature of the station multi-years |
|  | Hgh.avr.t | Monthly highest daily-average-temperature of the station multi-years |
|  | Lwr.avr.t | Monthly lowest daily-average-temperature of the station multi-years |
|  | Hgh.t | Monthly highest temperature of the station multi-years |
|  | Lwr.t | Monthly lowest temperature of the station multi-years |
|  | Avr.rt | Monthly average daily-range of temperature multi-years |
|  | Max.rt | Monthly maximum daily-range of temperature multi-years |
|  | Min.rt | Monthly minimum daily-range of temperature multi-years |
|  | Max.pt | Monthly maximum positive departure of temperature multi-years |
|  | Max.mt | Monthly maximum minus departure of temperature multi-years |
|  | Dt.g30 | Days in which the daily maximum temperature is greater than or equal to 30 degrees centigrade |
|  | Dt.g35 | Days in which the daily maximum temperature is greater than or equal to 35 degrees centigrade |
|  | Dt.g37 | Days in which the daily maximum temperature is greater than or equal to 37 degrees centigrade |
|  | Dt.g40 | Days in which the daily maximum temperature is greater than or equal to 40 degrees centigrade |
|  | Dt.l2 | Days in which the daily maximum temperature is less than or equal to 2 degrees centigrade |
|  | Dt.l0 | Days in which the daily maximum temperature is less than or equal to 0 degrees centigrade |
|  | Dt.lm2 | Days in which the daily maximum temperature is less than or equal to minus 2 degrees centigrade |
|  | Dt.lm15 | Days in which the daily maximum temperature is less than or equal to minus 15 degrees centigrade |
|  | Dt.lm30 | Days in which the daily maximum temperature is less than or equal to minus 30 degrees centigrade |
|  | Dt.lm40 | Days in which the daily maximum temperature is less than or equal to minus 40 degrees centigrade |
| Humidity | RH | Monthly average relative humidity multi-years |
| Precipitation | Avr.prc | Monthly average daily-precipitation multi-years |
|  | Max.prc | Monthly maximum precipitation multi-years |
|  | Min.prc | Monthly minimum precipitation multi-years |
|  | Max.d.prc | Monthly maximum daily-precipitation multi-years |
|  | Dp.lcp | Monthly precipitation of longest consecutive precipitation days multi-years |
|  | Max.cp | Monthly maximum of consecutive precipitation multi-years |
|  | Dp.g0.1 | Monthly days with daily precipitation greater than or equal to 0.1 mm multi-years |
|  | Dp.g1 | Monthly days with annual and daily precipitation greater than or equal to 1mm multi-years |
|  | Dp.g5 | Monthly days with daily precipitation greater than or equal to 5mm multi-years |
|  | Dp.g10 | Monthly days with daily precipitation greater than or equal to 10mm multi-years |
|  | Dp.g25 | Monthly days in which the daily precipitation in years is greater than or equal to 25mm multi-years |
|  | Dp.g50 | Monthly days with annual and daily precipitation greater than or equal to 50mm multi-years |
|  | Dp.g100 | Monthly days with annual and daily precipitation greater than or equal to 100mm multi-years |
|  | Dp.g150 | Monthly days in which the daily precipitation is greater than or equal to 150mm multi-years |
|  | Dp.lc | Monthly number of longest consecutive precipitation days multi-years |
|  | Dp.mxc | Monthly number of maximum consecutive precipitation days multi-years |
|  | Dwp.lc | The longest consecutive days without precipitation multi-years |
| Wind | Avr.ws | Monthly average wind speed multi-years |
|  | Max.ws | Maximum wind speed multi-years |
|  | Dws.g5 | Monthly days with the maximum daily wind speed is greater than or equal to 5m/s multi-years |
|  | Dws.g10 | Monthly days with the maximum daily wind speed is greater than or equal to 10m/s multi-years |
|  | Dws.g12 | Monthly days with the maximum daily wind speed is greater than or equal to 12m/s multi-years |
|  | Dws.g15 | Monthly days with the maximum daily wind speed is greater than or equal to 15m/s multi-years |
|  | Dws.g17 | Monthly days with the maximum daily wind speed is greater than or equal to 17m/s multi-years |

**Supplementary Table 2. Principal component analysis of meteorological factors**

| Meteorological factors | Variable | RC1 | RC2 | RC3 | h2 | u2 | com |
| --- | --- | --- | --- | --- | --- | --- | --- |
| Atmospheric pressure | Avr.p | 0.999 | 0.041 |  | 0.999 | 0.001 | 1.0 |
|  | Hgh.p | 0.999 | 0.033 |  | 1.000 | 0.000 | 1.0 |
|  | Lwr.p | 0.999 | 0.030 |  | 1.000 | 0.000 | 1.0 |
|  | Max.pp | 0.218 | 0.852 |  | 0.773 | 0.227 | 1.1 |
|  | Max.mp | -0.246 | -0.840 |  | 0.766 | 0.234 | 1.2 |
|  |  |  |  |  |  |  |  |
|  | SS loadings | 3.103 | 1.434 |  |  |  |  |
|  | Proportion Var | 0.621 | 0.287 |  |  |  |  |
|  | Cumulative Var | 0.621 | 0.907 |  |  |  |  |
|  | Proportion Explained | 0.684 | 0.316 |  |  |  |  |
|  | Cumulative Proportion | 0.684 | 1.000 |  |  |  |  |
| Temperature measurements | Avr.t | 0.976 | -0.204 | 0.048 | 0.996 | 0.004 | 1.1 |
|  | Hgh.avr.t | 0.981 | -0.062 | 0.149 | 0.989 | 0.011 | 1.1 |
|  | Lwr.avr.t | 0.932 | -0.349 | 0.066 | 0.994 | 0.006 | 1.3 |
|  | Hgh.t | 0.880 | -0.287 | -0.071 | 0.863 | 0.137 | 1.2 |
|  | Lwr.t | 0.904 | -0.153 | 0.356 | 0.967 | 0.033 | 1.4 |
|  | Avr.rt | -0.255 | 0.898 | 0.243 | 0.930 | 0.070 | 1.3 |
|  | Max.rt | -0.496 | 0.527 | 0.380 | 0.668 | 0.332 | 2.8 |
|  | Min.rt | -0.181 | 0.887 | 0.092 | 0.828 | 0.172 | 1.1 |
|  | Max.pt | -0.062 | -0.224 | -0.954 | 0.964 | 0.036 | 1.1 |
|  | Max.mt | 0.224 | 0.140 | 0.946 | 0.965 | 0.035 | 1.2 |
|  |  |  |  |  |  |  |  |
|  | SS loadings | 4.773 | 2.213 | 2.177 |  |  |  |
|  | Proportion Var | 0.477 | 0.221 | 0.218 |  |  |  |
|  | Cumulative Var | 0.477 | 0.699 | 0.916 |  |  |  |
|  | Proportion Explained | 0.521 | 0.241 | 0.238 |  |  |  |
|  | Cumulative Proportion | 0.521 | 0.762 | 1.000 |  |  |  |
| Temperature duration | Dt.g30 | -0.304 | 0.880 | 0.088 | 0.875 | 0.125 | 1.3 |
|  | Dt.g35 | -0.127 | 0.952 | 0.169 | 0.951 | 0.049 | 1.1 |
|  | Dt.g37 | -0.053 | 0.967 | 0.218 | 0.986 | 0.014 | 1.1 |
|  | Dt.g40 | 0.052 | 0.800 | 0.177 | 0.674 | 0.326 | 1.1 |
|  | Dt.l2 | 0.968 | -0.214 | 0.079 | 0.989 | 0.011 | 1.1 |
|  | Dt.l0 | 0.979 | -0.152 | 0.084 | 0.989 | 0.011 | 1.1 |
|  | Dt.lm2 | 0.981 | -0.099 | 0.082 | 0.979 | 0.021 | 1.0 |
|  | Dt.lm15 | 0.859 | 0.101 | 0.335 | 0.861 | 0.139 | 1.3 |
|  | Dt.lm30 | 0.221 | 0.240 | 0.928 | 0.968 | 0.032 | 1.3 |
|  | Dt.lm40 | 0.150 | 0.248 | 0.936 | 0.960 | 0.040 | 1.2 |
|  |  |  |  |  |  |  |  |
|  | SS loadings | 3.782 | 3.464 | 1.986 |  |  |  |
|  | Proportion Var | 0.378 | 0.346 | 0.199 |  |  |  |
|  | Cumulative Var | 0.378 | 0.725 | 0.923 |  |  |  |
|  | Proportion Explained | 0.410 | 0.375 | 0.215 |  |  |  |
|  | Cumulative Proportion | 0.410 | 0.785 | 1.000 |  |  |  |
| Precipitation measurements | Avr.prc | 0.958 | 0.244 |  | 0.978 | 0.022 | 1.1 |
|  | Max.prc | 0.841 | 0.532 |  | 0.989 | 0.011 | 1.7 |
|  | Min.prc | 0.951 | 0.005 |  | 0.905 | 0.095 | 1.0 |
|  | Max.d.prc | 0.162 | 0.971 |  | 0.970 | 0.030 | 1.1 |
|  | Dp.lcp | 0.913 | 0.314 |  | 0.932 | 0.068 | 1.2 |
|  | Max.cp | 0.830 | 0.515 |  | 0.955 | 0.045 | 1.7 |
|  |  |  |  |  |  |  |  |
|  | SS loadings | 4.079 | 1.650 |  |  |  |  |
|  | Proportion Var | 0.680 | 0.275 |  |  |  |  |
|  | Cumulative Var | 0.680 | 0.955 |  |  |  |  |
|  | Proportion Explained | 0.712 | 0.288 |  |  |  |  |
|  | Cumulative Proportion | 0.712 | 1.000 |  |  |  |  |
| Precipitation duration | Dp.g0.1 | 0.980 | 0.113 |  | 0.974 | 0.026 | 1.0 |
|  | Dp.g1 | 0.982 |  | 0.112 | 0.986 | 0.014 | 1.0 |
|  | Dp.g5 | 0.942 | 0.112 | 0.272 | 0.974 | 0.026 | 1.2 |
|  | Dp.g10 | 0.862 | 0.169 | 0.442 | 0.968 | 0.032 | 1.6 |
|  | Dp.g25 | 0.400 | 0.538 | 0.701 | 0.942 | 0.058 | 2.5 |
|  | Dp.g50 |  | 0.801 | 0.511 | 0.903 | 0.097 | 1.7 |
|  | Dp.g100 |  | 0.960 | 0.132 | 0.944 | 0.056 | 1.0 |
|  | Dp.g150 | 0.127 | 0.961 |  | 0.942 | 0.058 | 1.0 |
|  | Dp.lc | 0.951 |  | 0.174 | 0.935 | 0.065 | 1.1 |
|  | Dp.mxc | 0.934 |  | 0.228 | 0.925 | 0.075 | 1.1 |
|  | Dwp.lc | -0.833 | -0.158 | 0.211 | 0.764 | 0.236 | 1.2 |
|  |  |  |  |  |  |  |  |
|  | SS loadings | 6.208 | 2.867 | 1.182 |  |  |  |
|  | Proportion Var | 0.564 | 0.261 | 0.107 |  |  |  |
|  | Cumulative Var | 0.564 | 0.825 | 0.932 |  |  |  |
|  | Proportion Explained | 0.605 | 0.280 | 0.115 |  |  |  |
|  | Cumulative Proportion | 0.605 | 0.885 | 1.000 |  |  |  |
| Wind | Avr.ws | 0.102 | 0.449 | 0.817 | 0.880 | 0.120 | 1.6 |
|  | Max.ws | 0.542 | 0.194 | 0.728 | 0.861 | 0.139 | 2.0 |
|  | Dws.g5 | 0.938 | 0.174 | 0.144 | 0.930 | 0.070 | 1.1 |
|  | Dws.g10 | 0.814 | 0.477 | 0.297 | 0.978 | 0.022 | 1.9 |
|  | Dws.g12 | 0.709 | 0.615 | 0.324 | 0.985 | 0.015 | 2.4 |
|  | Dws.g15 | 0.458 | 0.819 | 0.333 | 0.992 | 0.008 | 1.9 |
|  | Dws.g17 | 0.221 | 0.906 | 0.320 | 0.972 | 0.028 | 1.4 |
|  |  |  |  |  |  |  |  |
|  | SS loadings | 2.606 | 2.366 | 1.625 |  |  |  |
|  | Proportion Var | 0.372 | 0.338 | 0.232 |  |  |  |
|  | Cumulative Var | 0.372 | 0.710 | 0.942 |  |  |  |
|  | Proportion Explained | 0.395 | 0.359 | 0.246 |  |  |  |
|  | Cumulative Proportion | 0.395 | 0.754 | 1.000 |  |  |  |

**Supplementary Table 3. Number of epidemic counties and classification margins at different levels**

| Level | County No. | ln(pr).min | ln(pr).max | Margin of pr(%) |
| --- | --- | --- | --- | --- |
| 1 | 3 | -6.53 | -5.16 | ~0.01 |
| 2 | 22 | -3.94 | -2.97 | ~0.05 |
| 3 | 16 | -2.87 | -1.97 | ~0.15 |
| 4 | 29 | -1.81 | -1.00 | ~0.38 |
| 5 | 24 | -0.92 | 0.319 | ~1.73 |
| 6 | 5 | 0.73 | 2.72 | 1.73~ |

**Supplementary Table 4. Linear regression model**

| Model | Dependent variable | Independent variable | Coefficients | | | | |
| --- | --- | --- | --- | --- | --- | --- | --- |
|  |  |  | Estimate | Std. Error | t value | Pr(>\|t\|) | Signif. codes |
| Multivariable Linear Regression Model | ln(pr) | (Intercept) | -3.3233 | 7.8895 | -0.4210 | 0.6746 |  |
|  |  | atmos_anom | -0.0313 | 0.0560 | -0.5590 | 0.5778 |  |
|  |  | temp_Val | -0.0204 | 0.0579 | -0.3520 | 0.7256 |  |
|  |  | temp_anom | 0.0626 | 0.0916 | 0.6830 | 0.4961 |  |
|  |  | CldD | 0.0426 | 0.0748 | 0.5690 | 0.5707 |  |
|  |  | ExtrHtD | 0.1978 | 0.1066 | 1.8560 | 0.0668 |  |
|  |  | prec_Val_M | 0.0094 | 0.0025 | 3.7430 | 0.0003 | *** |
|  |  | Fsh_brz_Ds | -0.0429 | 0.0586 | -0.7320 | 0.4662 |  |
|  |  | DEM | 0.0001 | 0.0003 | 0.4190 | 0.6766 |  |
| Stepwise Regression Model | ln(pr) | (Intercept) | -1.2789 | 2.4063 | -0.5320 | 0.5963 |  |
|  |  | atmos_anom | -0.0632 | 0.0226 | -2.7980 | 0.0062 | ** |
|  |  | CldD | 0.0762 | 0.0155 | 4.9190 | 0.0000 | *** |
|  |  | ExtrHtD | 0.2191 | 0.0913 | 2.3990 | 0.0184 | * |
|  |  | prec_Val_M | 0.0093 | 0.0023 | 4.0110 | 0.0001 | *** |

Note: *** p<0.001; ** p<0.01; * p<0.05

**Supplementary Table 5. Multinomial logistic regression model**

| Level Class | (Intercept) | atmos_anom | temp_Val | temp_anom | CldD | ExtrHtD | prec_Val_M | Fsh_brz_Ds | DEM |
| --- | --- | --- | --- | --- | --- | --- | --- | --- | --- |
| 2 | -39.3689*** | 2.2768*** | -4.8089*** | 2.1076*** | -3.4755*** | 10.4867*** | 0.6094*** | 0.3693* | 0.0053*** |
| 3 | 45.2812*** | 1.6139*** | -4.979*** | 1.6031*** | -3.6033*** | 11.157*** | 0.5941*** | 0.1686NotSignif | 0.0041** |
| 4 | 42.5262*** | 1.5981*** | -4.9917*** | 1.5165*** | -3.4877*** | 11.4873*** | 0.6049*** | 0.1547NotSignif | 0.0044** |
| 5 | -56.7733*** | 2.4853*** | -5.2862*** | 2.6952*** | -3.965*** | 10.8812*** | 0.6269*** | 0.1751NotSignif | 0.0096*** |
| 6 | 0.8472*** | -0.9358*** | -3.3586*** | 5.7915*** | -1.6223*** | 18.3617*** | 0.8187*** | -1.1054*** | 0.0091NotSignif |

Note: Reference class is Level 1, *** p<0.001, ** p<0.01, * p<0.05, NotSignif p≥0.05

**Supplementary Table 6. Naive Bayesian classification model**

| Prior probabilities | Levels | 1 | 2 | 3 | 4 | 5 | 6 |
| --- | --- | --- | --- | --- | --- | --- | --- |
|  | Weighted | 0.0303 | 0.2222 | 0.1616 | 0.2929 | 0.2424 | 0.0505 |
| Coefficients of linear discriminants | Independent variable | LD1 | LD2 | LD3 | LD4 | LD5 |  |
|  | atmos_anom | 0.0259 | -0.3047 | 0.1750 | -0.1720 | 0.0951 |  |
|  | temp_Val | 0.0563 | -0.0685 | -0.2994 | 0.1294 | 0.1635 |  |
|  | temp_anom | -0.1437 | -0.1352 | -0.0344 | -0.6191 | 0.3974 |  |
|  | CldD | 0.0036 | -0.1329 | -0.4099 | 0.3083 | 0.0307 |  |
|  | ExtrHtD | -0.2816 | 0.2721 | -0.2978 | -0.0537 | -0.4499 |  |
|  | prec_Val_M | -0.0109 | -0.0138 | -0.0057 | 0.0056 | 0.0035 |  |
|  | Fsh_brz_Ds | 0.0514 | -0.0595 | 0.1327 | -0.0686 | -0.0233 |  |
|  | DEM | -0.0002 | -0.0004 | 0.0013 | -0.0003 | -0.0004 |  |

**Supplementary Figure 1. Correlations between the natural logarithm of the prevalence rate of alveolar echinococcosis and characteristic indicators**

Note: The blue line represents the single-factor regression curve, the region between the red lines represents the prediction interval, and the grey area represents the 95% confidence interval.

**Supplementary Figure 2. Mapping of ln(pr) and effective composite indicators**

Note: A ~ P represents Mapping of atmos_an, atoms_Val, temp_anom, temp_Val, CldD, ExtrHtD, ExtrCldD, prec_Val_M, prec_Val_D, Rainy_Ds, raintorm_Ds, Gt_brz_Ds, Fsh_brz_Ds, Wnd_spd_val, DEM, ln(pr).

**Supplementary Figure 3. Results of the 10-fold cross-validation**

**Supplementary Figure 4. The three-level indicator system**

**Supplementary Figure 5. Selection of classification number**

**Supplementary Figure 6. Classification and Regression Tree Model**
