# Supplementary figures and images for: Developing a geographical–meteorological indicator system and evaluating prediction models for alveolar echinococcosis in China
Source: J Expo Sci Environ Epidemiol. 2024 Apr 23;35(2):254–63. doi: 10.1038/s41370-024-00664-z (PMC12009731; doi:10.1038/s41370-024-00664-z)

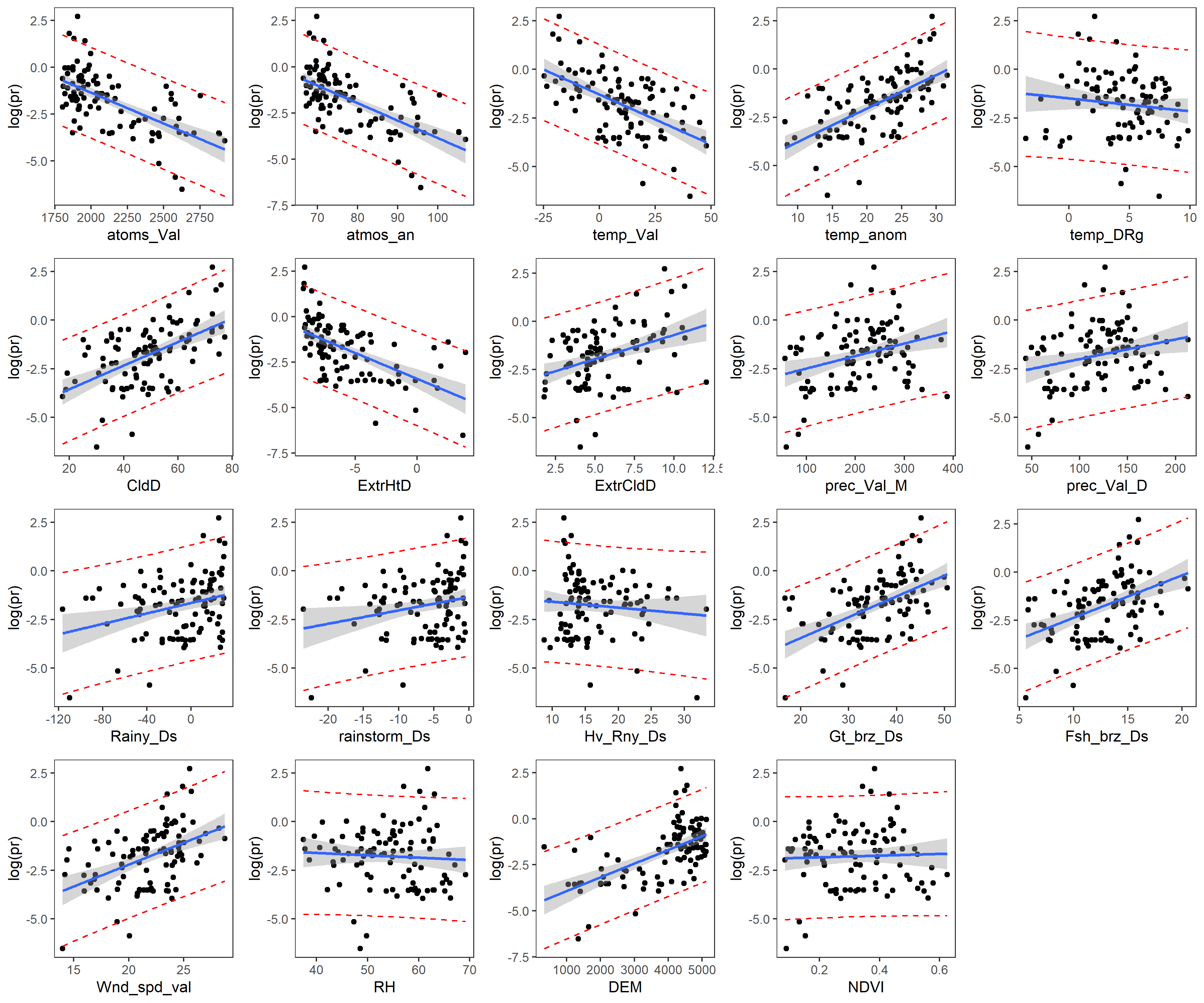

Supplement: Supplementary file 2 — Supplementary Figure 1 [file 41370_2024_664_MOESM2_ESM.tif]

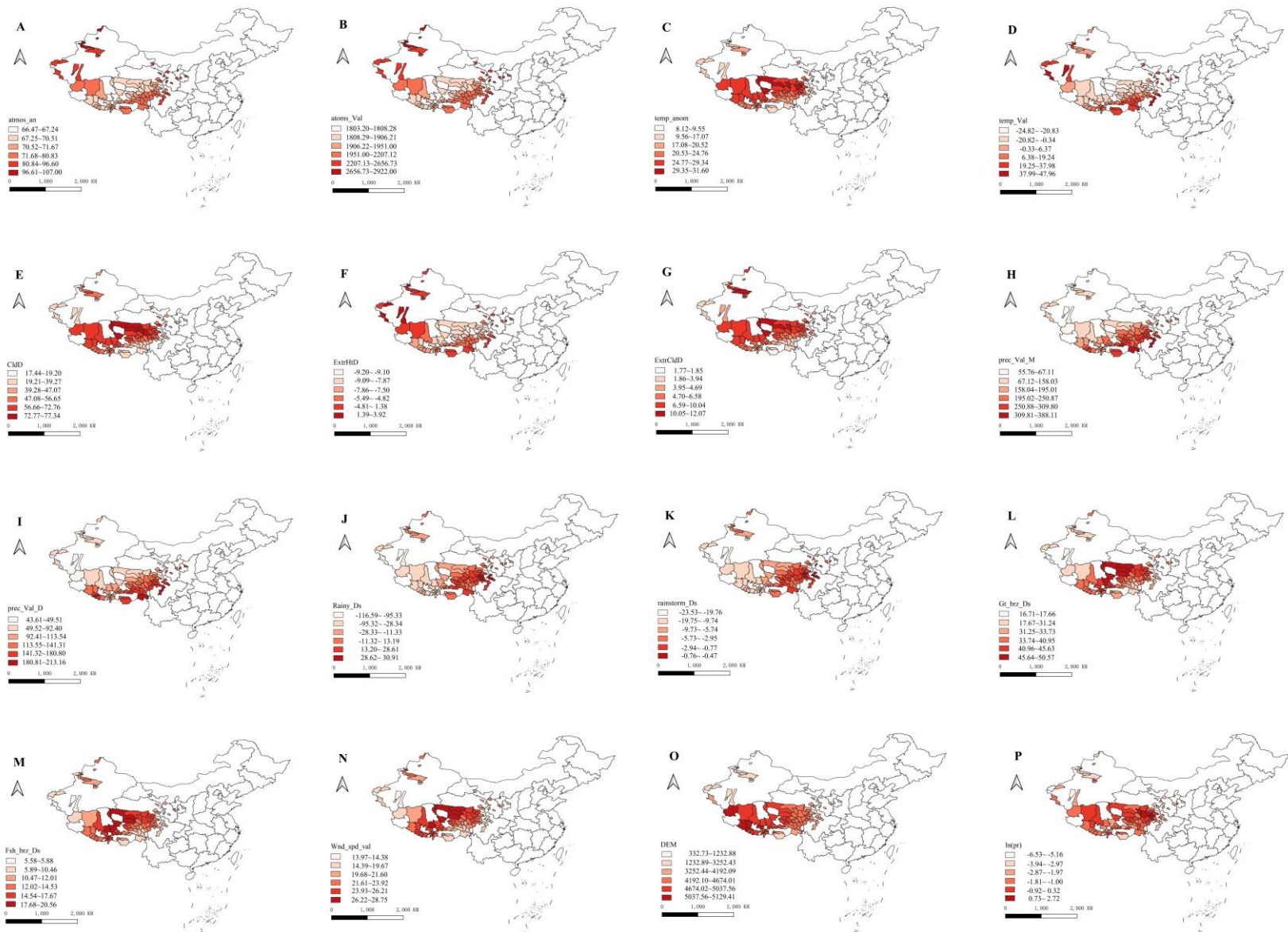

Supplement: Supplementary file 3 — Supplementary Figure 2 [file 41370_2024_664_MOESM3_ESM.pdf]

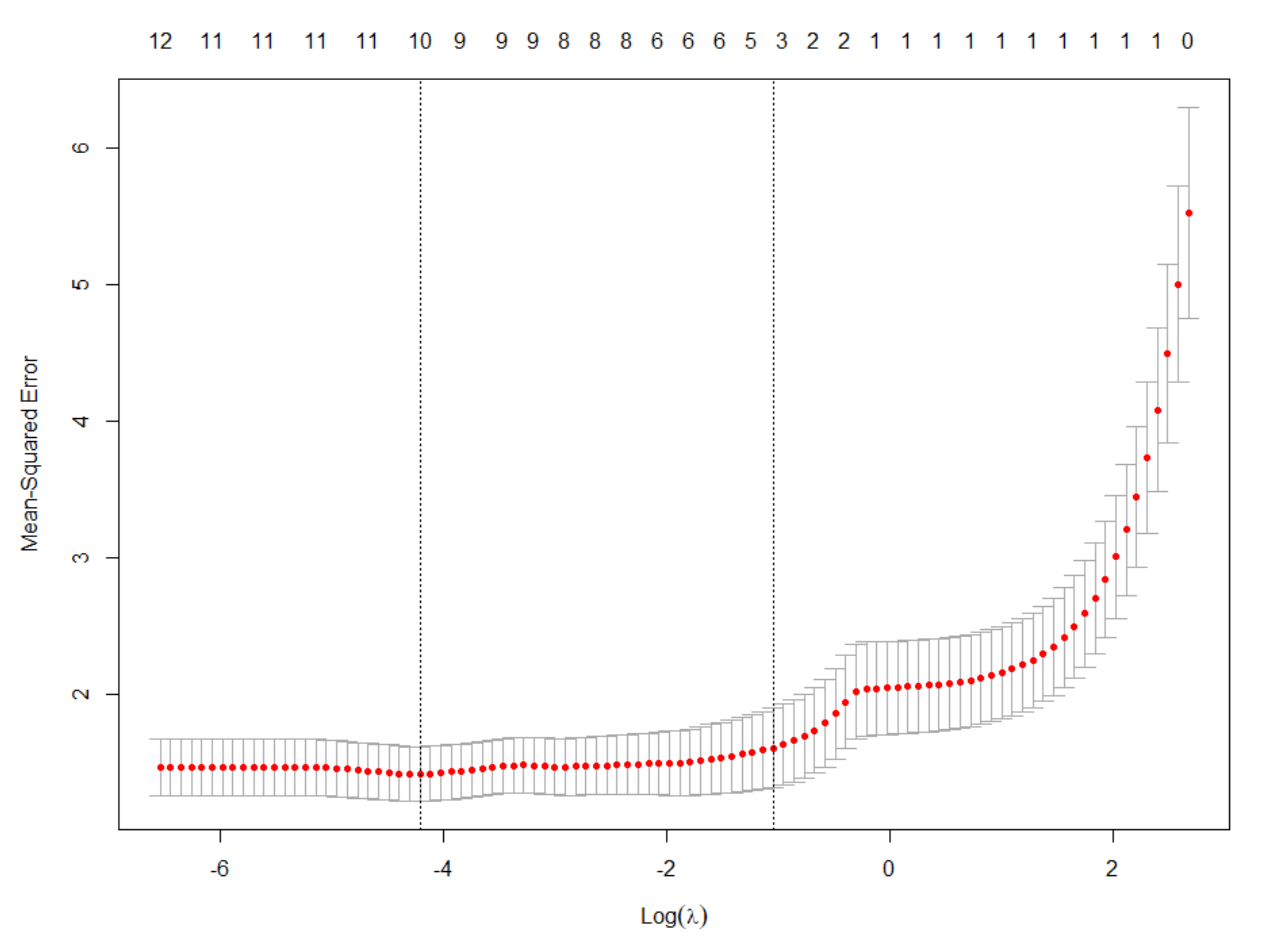

Supplement: Supplementary file 4 — Supplementary Figure 3 [file 41370_2024_664_MOESM4_ESM.tif]

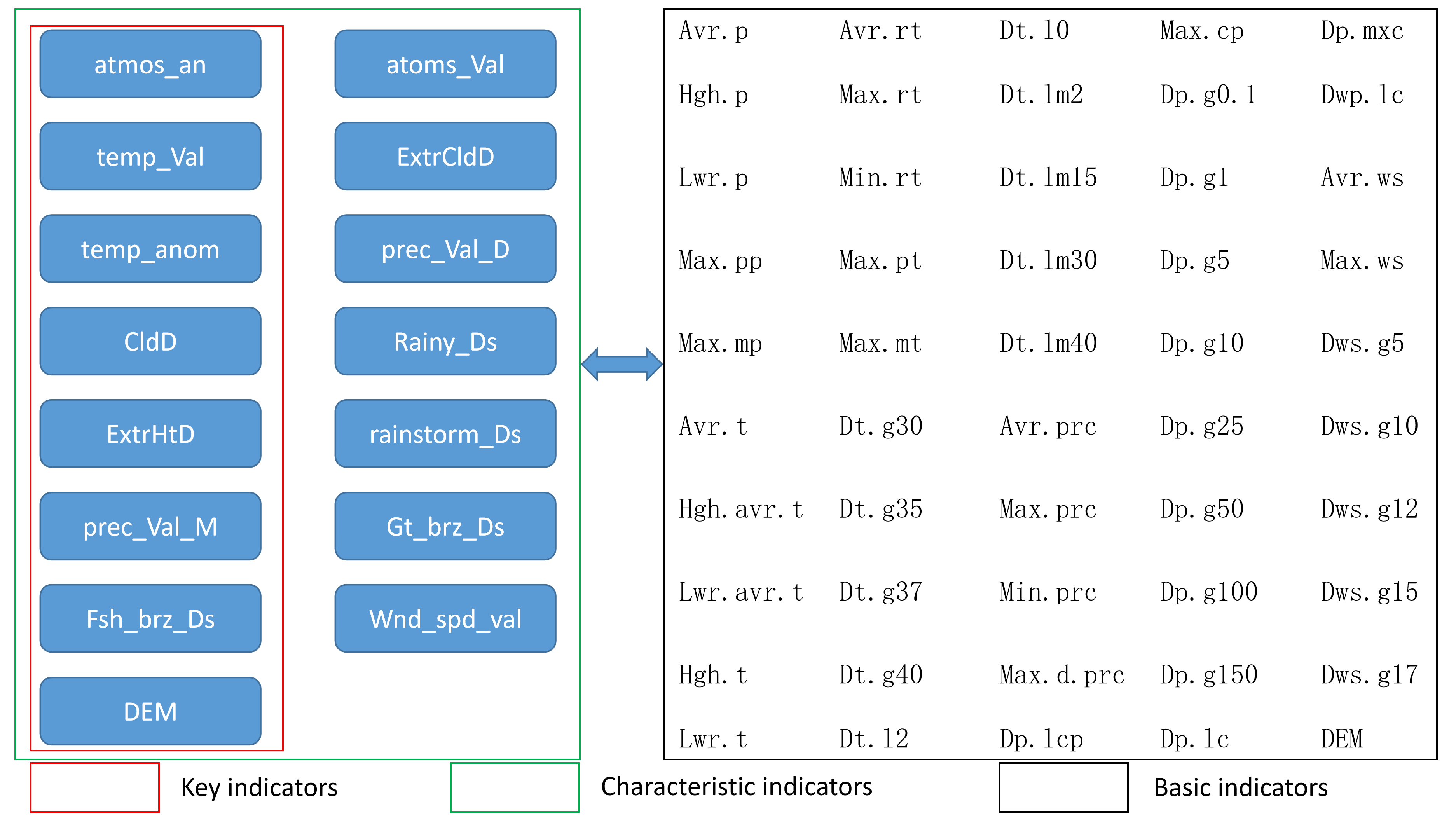

Supplement: Supplementary file 5 — Supplementary Figure 4 [file 41370_2024_664_MOESM5_ESM.tif]

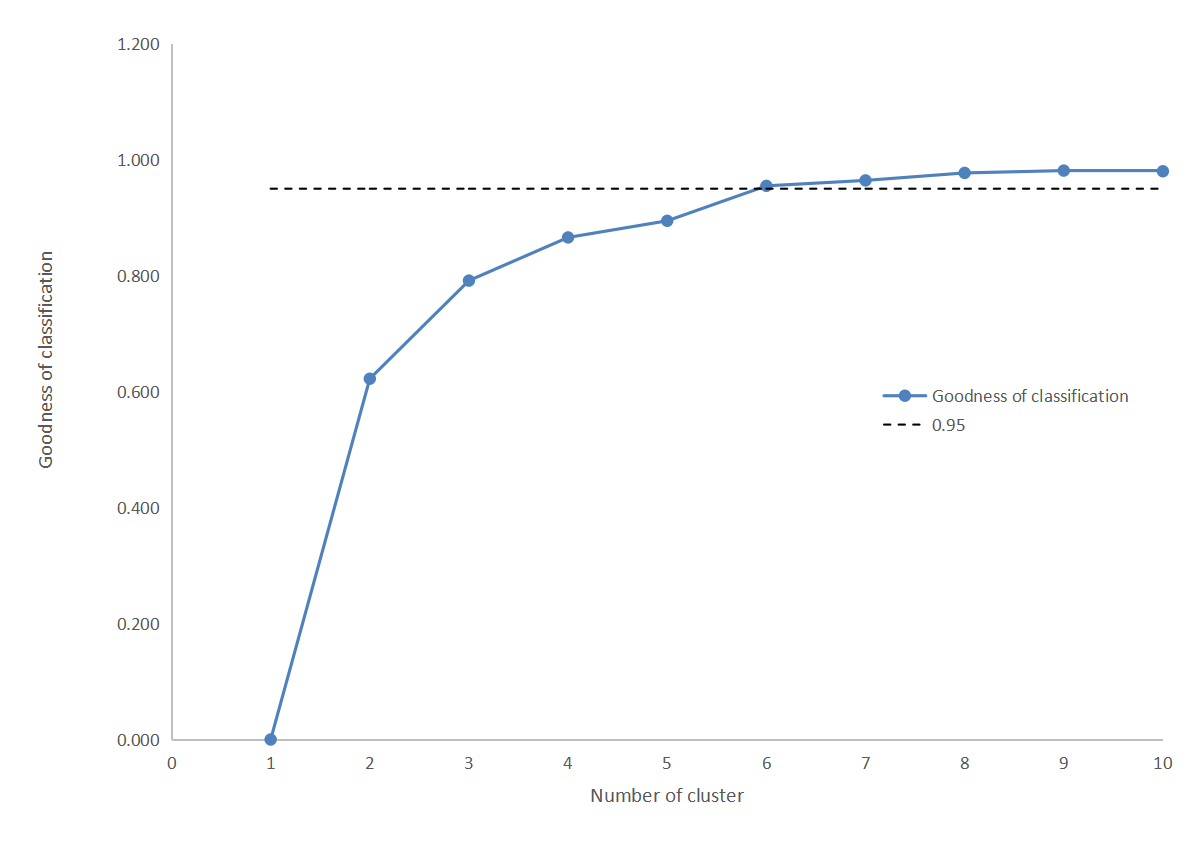

Supplement: Supplementary file 6 — Supplementary Figure 5 [file 41370_2024_664_MOESM6_ESM.tif]

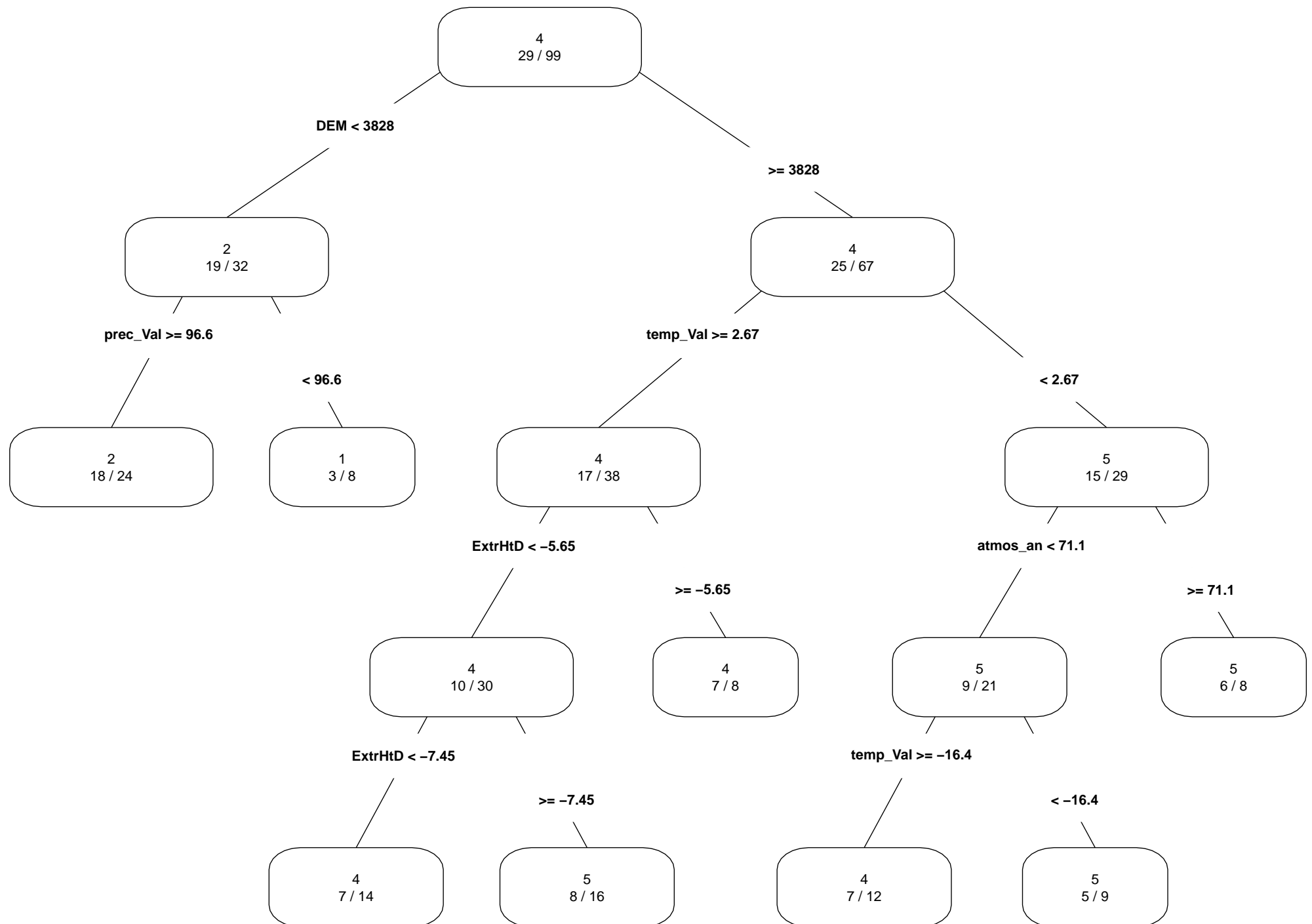

Supplement: Supplementary file 7 — Supplementary Figure 6 [file 41370_2024_664_MOESM7_ESM.pdf]
